# Therapeutic Exosomes Carrying *VEGFA* siRNA Inhibit Pathological Corneal Angiogenesis via PI3K–Akt–Caspase-3 Signaling

**DOI:** 10.3390/biomedicines14010246

**Published:** 2026-01-21

**Authors:** Woojune Hur, Basanta Bhujel, Seorin Lee, Seheon Oh, Ho Seok Chung, Hun Lee, Jae Yong Kim

**Affiliations:** 1 Department of Ophthalmology, Asan Medical Center, University of Ulsan College of Medicine, Seoul 05505, Republic of Korea; dnwnsgj@naver.com (W.H.); basantabhujel86@gmail.com (B.B.);; 2 Department of Medical Science, University of Ulsan Graduate School, Seoul 05505, Republic of Korea

**Keywords:** mesenchymal stem cell (MSC), exosomes, VEGFA, corneal neovascularization, phosphoinositide 3-kinase (PI3K)/protein kinase B (Akt) pathway, caspase-3, apoptosis, gene therapy, anti-angiogenesis

## Abstract

**Background/Objectives:** Neovascularization, defined as the sprouting of new blood vessels from pre-existing vasculature, is a critical pathological feature in ocular diseases such as pathological myopia and represents a leading cause of corneal vision loss. Vascular endothelial growth factor A (VEGFA) plays a pivotal role in endothelial cell proliferation, migration, survival by anti-apoptotic signaling, and vascular permeability. Dysregulation of VEGFA is closely linked to pathological neovascularization. Exosomes, nanosized phospholipid bilayer vesicles ranging from 30 to 150 nm, have emerged as promising gene delivery vehicles due to their intrinsic low immunogenicity, superior cellular uptake, and enhanced in vivo stability. This study aimed to investigate whether highly purified mesenchymal stem cell (MSC)-derived exosomes loaded with *VEGFA* siRNA labeled with FAM can effectively suppress pathological corneal neovascularization (CNV) via targeeted cellular transduction and VEGFA inhibition. Furthermore, we examined whether the therapeutic effect involves the modulation of the PI3K–Akt–Caspase-3 signaling axis. **Methods:** Exosomes purified by chromatography were characterized by electronmicroscopy, standard marker immunoblotting, and nanoparticle tracking analysis. In vitro, we assessed exosome uptake and cytoplasmic release, suppression of *VEGFA* mRNA/protein, cell viability, and apoptosis. In a mouse CNV model, we evaluated tissue reach and stromal retention after repeated intrastromal injections; anterior segment angiogenic indices; CD31/VEGFA immunofluorescence/immunoblotting; phosphorylated PI3K and Akt; cleaved caspase-3; histology (H&E); and systemic safety (liver, kidney, and spleen). **Results:** Exosomes were of high quality and showed peak efficacy at 48 h, with decreased *VEGFA* mRNA/protein, reduced viability, and increased apoptosis in vitro. In vivo, efficient delivery and stromal retention were observed, with accelerated inhibition of neovascularization after Day 14 and maximal effect on Days 17–19. Treatment reduced CD31 and VEGFA, decreased p-PI3K and p-Akt, and increased cleaved caspase-3. Histologically, concurrent reductions in neovascularization, inflammatory cell infiltration, and inflammatory epithelial thickening were observed, alongside a favorable systemic safety profile. **Conclusions:** *VEGFA* siRNA-loaded exosomes effectively reduce pathological CNV via a causal sequence of intracellular uptake, cytoplasmic release, targeted inhibition, and phenotypic suppression. Supported by consistent PI3K–Akt inhibition and caspase-3–mediated apoptosis induction, these exosomes represent a promising local gene therapy that can complement existing antibody-based treatments.

## 1. Introduction

Neovascularization, which involves the formation of new blood vessels from existing vasculature, is a hallmark of several vision-threatening ocular diseases, including pathological myopia, diabetic retinopathy, age-related macular degeneration, retinal vein occlusion, and corneal neovascularization (CNV) [1,2]. It is a primary cause of central vision loss [1,2]. Current treatments, such as intraocular injections of vascular endothelial growth factor (VEGF) inhibitors, have demonstrated significant clinical benefits, especially in cases of diabetic retinopathy and macular degeneration [3,4]. Among VEGF isoforms, *VEGFA* is a crucial regulator of angiogenesis. It promotes endothelial cell proliferation and migration, inhibits apoptosis, and promotes vascular growth and permeability [5,6,7]. Abnormal activation of VEGFA is strongly associated with pathological neovascularization in various diseases [5]. Exosomes are nanoscale vesicles (30–150 nm in diameter) enclosed by a phospholipid bilayer and secreted by various cell types. They carry diverse molecular cargo, including proteins, lipids, RNAs, and DNA, and facilitate intercellular communication [8,9]. Due to their membrane structure, exosomes exhibit efficient cellular uptake, and when derived from autologous sources, they have low immunogenicity and high stability in vivo, making them promising vehicles for therapeutic delivery [10,11].

Recent studies have highlighted the potential of exosomes as carriers for gene-silencing agents such as small interfering RNAs (siRNAs) [12]. Building on this, we investigated whether mesenchymal stem cell (MSC)-derived exosomes loaded with *VEGFA* siRNA could inhibit pathological angiogenesis by downregulating VEGFA expression at both the mRNA and protein levels. We also assessed whether this therapeutic effect involved modulation of the PI3K–Akt–caspase-3 pathway, a signaling cascade known to affect endothelial cell survival and apoptosis. Our research was guided by three main hypotheses. First, exosomes loaded with *VEGFA* siRNA would efficiently enter target cells, degrade *VEGFA* mRNA, and inhibit angiogenesis by downregulating VEGFA and other pro-angiogenic factors. Second, exosome-based delivery would improve safety and effectiveness compared to current therapies, due to better cellular uptake and efficient release into the cytoplasm. Third, at the molecular level, blocking VEGFA signaling would reduce CNV by inhibiting the PI3K–Akt pathway, a crucial endothelial survival pathway, leading to the activation of caspase-3-mediated apoptosis and destabilization of abnormal new vessels.

To this end, we developed an exosome-based *VEGFA* siRNA delivery platform and examined its cellular uptake, gene silencing effectiveness, and pro-apoptotic activity in vitro. Then, we tested its therapeutic potential in a mouse model of corneal neovascularization and explored the underlying mechanism, focusing on the modulation of the PI3K–Akt–caspase-3 signaling cascade. These results show that exosome-mediated *VEGFA* silencing is a promising approach for reducing pathological CNV and could lay the groundwork for future targeted, gene-based ocular treatments.

## 2. Materials and Methods

### 2.1. Cell Culture

Human MSCs derived from umbilical cord tissue (PCS-500-010, ATCC, Manassas, VA, USA) and human umbilical vein endothelial cells (HUVECs; PCS-100-010, ATCC) were used in this study. MSCs were cultured in Minimum Essential Medium Alpha (MEM Alpha; 12571-063, Gibco, Thermo Fisher Scientific Inc., Waltham, MA, USA) supplemented with 10% fetal bovine serum (FBS; 10099-141, Gibco) and 1% antibiotic–antimycotic solution (15240-062, Gibco). HUVECs were cultured in Medium 200 (M200500, Gibco), a basal medium for large-vessel endothelial cells, supplemented with low-serum growth supplement (LSGS; S00310, Gibco), 10% FBS, and 1% antibiotic–antimycotic solution. All cells were maintained at 37 °C in a humidified incubator with 5% CO_2_ and 95% air.

### 2.2. Exosome Purification

Exosomes were isolated from MSC-conditioned medium using a high-purity multi-column chromatography protocol, as previously described [13]. Briefly, MSCs were seeded in 150 cm^2^ culture dishes and maintained for 5 days in MEM Alpha medium without FBS. On the day of harvest, the conditioned medium was collected and centrifuged at 10,000× *g* for 30 min at 4 °C to remove cellular debris. The supernatant was retained, and a 0.5 mL aliquot was loaded onto a pre-equilibrated chromatography column. After the sample passed through the first chromatography disk, phosphate-buffered saline (PBS) was added to the column as an elution buffer. The eluate was collected in 0.5 mL fractions, yielding a total of 13 fractions. Fractions 11 and 12, identified to contain purified exosomes based on prior validation, were pooled and concentrated to a final volume of 100 μL using centrifugal filtration (Amicon Ultra-0.5 mL, Millipore, Burlington, MA, USA) according to the manufacturer’s instructions.

### 2.3. Transmission Electron Microscopy (TEM)

Exosomes were visualized by TEM using an HT7800 system (Hitachi, Tokyo, Japan) equipped with a DC-heated tungsten filament electron gun and operated at an acceleration voltage of 100 kV (maximum 120 kV). Images were captured with an AMT Nanosprint camera (Woburn, MA, USA), achieving a resolution of 0.20 nm at an acceleration voltage of 100 kV. The instrument provided magnifications from ×200 to ×200,000 in high-contrast mode and from ×4000 to ×600,000 in high-resolution mode. An LN_2_ cold trap was used as needed. Negative staining was performed for exosome visualization. TEM grids were first rendered hydrophilic via glow discharge (SMC12R-Plus, Semian Technology, Cheonan-City, Republic of Korea) for 10–15 s. A droplet of exosome suspension was applied to the grid, and excess fluid was removed with filter paper. The grid was then stained with 1% uranyl acetate, followed by blotting and air drying prior to imaging.

### 2.4. Nanoparticle Tracking Analysis (NTA)

Particle size distribution and concentration of exosomes were measured using NTA on a NanoSight LM10 system (Malvern Panalytical Ltd., Malvern, UK) equipped with a 40 mW 642 nm red laser and CCD camera. Data were acquired and analyzed using NanoSight NTA 3.4 software. The instrument has a particle size detection range of approximately 10–40 nm (<d < 1–2 μm), with a practical lower limit of approximately 60 nm for biological particles, such as exosomes. The quantifiable concentration range was approximately 10^6^ to 10^9^ particles/mL. The sample temperature was controlled within a range of ~5 °C below ambient to 50 °C.

### 2.5. Loading of VEGFA siRNA-FAM into Exosomes

*VEGFA* siRNA labeled with fluorescein amidite (FAM) was loaded into MSC-derived exosomes using the Exo-Fect transfection reagent (System Biosciences, Palo Alto, CA, USA) to generate the exosome delivery platform. Purified exosomes were first resuspended in 500 μL of sterile 1× PBS. To this, 10 μL of Exo-Fect reagent, 20 μL of *VEGFA* siRNA-FAM (20 pmol; Table 1), 70 μL of PBS, and 50 μL of purified exosomes (1 × 10^6^ particles) were sequentially added and mixed thoroughly. The mixture was incubated on ice at 4 °C for 30 min. Following incubation, the transfection reaction was centrifuged at 14,000 rpm for 3 min. The supernatant was discarded, and the pellet with transduced exosomes was resuspended in 300 μL of sterile PBS.

### 2.6. Western Blot Analysis

For protein extraction and Western Blot analysis, exosome samples (including un-loaded and *VEGFA* siRNA-FAM-loaded exosomes for in vitro studies) and corneal tis-sues (for in vivo studies) were lysed in an extraction buffer (Intron Biotech, Inc., Seoul, Re-public of Korea) containing 1× RIPA buffer, consisting of 40 mM Tris-HCl (pH 7.4), 1% Triton X-100, 0.1% SDS, 150 mM NaCl, 10% glycerol, 1 mM EDTA, 50 mM NaF, 1 mM PMSF, 1 mM Na_3_VO_4_, 5 mM dithiothreitol, and protease inhibitors (leupeptin, pepstatin, and aprotinin, each at 1 μg/mL). Lysates were centrifuged at 13,000× *g* for 15 min at 4 °C, and the supernatants were collected. Protein concentrations were measured using the Pierce™ BCA Protein Assay Kit (Thermo Fisher Scientific Inc.).

Equal amounts of protein (50–80 μg) were separated on 12% SDS-polyacrylamide gels and transferred to PVDF membranes. Membranes were washed with Tris-buffered saline containing 0.1% Tween-20 (TBST) and blocked with 5% nonfat dry milk in TBST for 1 h at room temperature. Membranes were then incubated overnight at 4 °C with primary antibodies (Table 2), followed by incubation with secondary antibodies (Table 2). Protein bands were visualized using enhanced chemiluminescence (ECL) reagents (Santa Cruz Biotechnology, Dallas, TX, USA), and images were captured on X-ray film after exposure to a chemiluminescent substrate (Merck Millipore, Burlington, MA, USA).

### 2.7. Transfection of VEGFA siRNA-FAM-Loaded Exosomes into Target Cells

To enhance the intracellular delivery of *VEGFA* siRNA-FAM, the EV-Entry system (System Biosciences, Palo Alto, CA, USA) was used. This system promotes efficient exosome uptake and cytoplasmic internalization, consisting of two reagents (A and B) that are mixed with exosomes immediately before transfection. Briefly, the purified exosome pellet was resuspended in 93 μL of sterile Dulbecco’s Modified Eagle Medium (DMEM; serum- and antibiotic-free; Gibco). To this suspension, 5 μL of 20× EV-Entry Reagent A and 2 μL of 1× EV-Entry Reagent B were added sequentially, corresponding to a total of 1 × 10^6^ exosome particles. The mixture was gently pipetted three times and incubated at room temperature for 45 min to allow transfection prior to application to target cells.

### 2.8. RT-PCR Analysis

To assess *VEGFA* mRNA knockdown following exosomal delivery of siRNA, total RNA was extracted from target cells and reverse-transcribed into complementary DNA (cDNA). One microgram of total RNA was used for cDNA synthesis using the First Strand cDNA Synthesis Kit (Thermo Fisher Scientific Inc.). RNA concentration and purity were determined by absorbance at 260 nm using a NanoDrop 2000 spectrophotometer (Thermo Fisher Scientific Inc.). Reverse transcription-polymerase chain reaction (RT-PCR) was performed using oligo(dT)18 primers on a C-1000 Touch thermal cycler (Bio-Rad Laboratories, Inc., Hercules, CA, USA). PCR amplification was carried out using Taq DNA polymerase (Thermo Fisher Scientific Inc.) and gene-specific primers (Table 1). The thermal cycling conditions were as follows: initial denaturation at 95 °C for 5 min, followed by 38 cycles of 95 °C for 30 s, 55–60 °C for 30 s (primer-dependent), and 72 °C for 1 min, with a final extension step at 72 °C for 7 min. PCR products were stored at 4 °C until further analysis.

### 2.9. Multiplex Assay

Multiplex immunoassays were performed using the Bio-Plex multiplex system (Bio-Rad Laboratories, Inc.), based on Luminex xMAP bead-based technology, according to the manufacturer’s instructions. VEGFA cytokine concentrations were measured in supernatants collected from human MSC cultures. Briefly, 50 μL of diluted magnetic bead (microparticle) cocktail was added to each well of a 96-well plate and incubated for 2 h at room temperature with constant agitation (800 rpm). Following this, 50 μL of standard or sample and 50 μL of diluted biotinylated detection antibody cocktail were added to each well. The plates were sealed and incubated for 1 h at room temperature under the same agitation conditions.

After washing, 50 μL of diluted streptavidin–phycoerythrin (streptavidin-PE) was added, and the plate was incubated for an additional 30 min at room temperature with agitation at 800 rpm. Following a second wash step, residual liquid was removed, and 100 μL of wash buffer was added and removed again. A final 100 μL of wash buffer was then added to each well, and plates were read within 90 min using either a Luminex analyzer (Thermo Fisher Scientific Inc.) or a Bio-Plex reader (Bio-Rad Laboratories, Inc.). Cytokine concentrations were quantified using Bio-Plex Manager software (Bio-Plex Manager 6.2 software).

### 2.10. Fluorescence-Activated Cell Sorting (FACS) Assay

To evaluate apoptosis induced by *VEGFA* siRNA-FAM-loaded exosomes, flow cytometry was performed using Annexin V-fluorescein isothiocyanate (FITC) and propidium iodide (PI) staining. Cells from each experimental group were harvested and incubated with Annexin V–FITC and PI (Thermo Fisher Scientific Inc.) for 30 min at room temperature in the dark. FITC- and PE-conjugated isotype control immunoglobulin (Ig)G antibodies (Thermo Fisher Scientific Inc.) were used as negative staining controls. After staining, cells were washed twice with FACS buffer, resuspended in fresh buffer, and analyzed using a FACScan flow cytometer (Becton Dickinson and Company, Franklin Lakes, NJ, USA).

### 2.11. Tube Formation Assay

To assess the anti-angiogenic effect of *VEGFA* siRNA-FAM-loaded exosomes, a tube formation assay was conducted. HUVECs were seeded at a density of 10,000 cells per well in 24-well plates (SPL Life Sciences, Pocheon-si, Gyeonggi-do, Republic of Korea), precoated with 1 mL of Matrigel (Corning, One Riverfront Plaza, Corning, NY, USA) per well. The cells were cultured in complete growth medium containing exosome-depleted fetal bovine serum (FBS; Gibco) at 37 °C. After 24 h, tube-like structures were confirmed microscopically, and the medium was replaced with serum-free medium. On the following day, *VEGFA* siRNA-FAM-loaded exosomes (100 μL/mL) were added to the treatment groups at specified time points. Tube formation was visualized using a Leica phase-contrast microscope, and images were captured for analysis. The number of tubes per well was quantified using ImageJ 1.54g software (National Institutes of Health, Bethesda, MD, USA). All experiments were performed independently and repeated three times.

### 2.12. Confocal Microscopy

Confocal microscopy was used to evaluate the intracellular localization of exosomes loaded with FAM-labeled *VEGFA* siRNA (green fluorescence) and to visualize immuno-fluorescence staining of both in vitro and in vivo samples. For in vitro imaging, target cells were seeded into four-well culture plates and treated with labeled exosomes the following day. At the indicated time points, cells were fixed with 4% paraformaldehyde and blocked with a solution of 3% FBS and 1% bovine serum albumin (BSA) for 1 h at room temperature. After, nuclei were stained with DAPI (1:10,000; Invitrogen, Thermo Fisher Scientific Inc.) for 10 min.

For in vivo immunostaining, paraffin-embedded tissue sections were deparaffinized by two 10-min incubations in xylene, followed by sequential rehydration in 100% ethanol (2 × 5 min), 95% ethanol (1 × 10 min), and 70% ethanol (1 × 5 min). Sections were then rinsed in distilled water for 5 min to complete rehydration. Antigen retrieval was performed by boiling the sections in a 50 mM dissolving gel solution for 30 min, then cooling at room temperature for 30 min. Sections were first blocked with 3% BSA for 1 h at room temperature to prevent nonspecific binding and then incubated with primary antibodies (Table 2) diluted in 3% BSA. After three PBS washes, corresponding secondary antibodies diluted in PBS with 3% BSA were applied and incubated for 1 h. Nuclear staining was performed using DAPI (1:1000) for 10 min, followed by PBS washes and mounting with antifade mounting medium. Fluorescence images were taken using a confocal laser scanning microscope (LSM, Carl Zeiss AG, Oberkochen, Germany) and processed with ImarisViewer software (version 10.0, Oxford Instruments plc, Abingdon, UK).

### 2.13. Animals

Eight-week-old female BALB/c mice (BALB/cAnTacDbl strain, BL Co., Ltd., Pyeongtaek, Gyeonggi, Republic of Korea) with an average weight of 19 g were housed under specific pathogen-free conditions. Animals were provided with a standard pellet diet (LAB RODENT CHOW, Cargill Agri Purina Inc., Shoreview, MN, USA) and sterile water ad libitum. The bedding consisted of GLP Aspen bedding (wood chips, ABEDD, Kalnciems, Jelgavas nov., Latvia), and the cages used were Blue line (Tecniplast, Buguggiate, Italy) with a floor area of 542 cm^2^ (84 in^2^). Enrichment materials included NESTLETS BY ANCARE (nesting material, ANCARE, Bellmore, NY, USA) and wood bricks (Jeongdo B&P, Gwangju-si, Gyeonggi-do, Republic of Korea; 50 mm × L10 mm × H10 mm). Mice were kept at a controlled temperature of 22 ± 2 °C, relative humidity of 40–60%, and a 12-h light/dark cycle. A minimum acclimation period of 7 days was observed before experimentation. Mice were randomly assigned to experimental groups after individual identification. All outcome assessments were performed blinded. Anesthesia was given via isoflurane inhalation or intraperitoneal injection of ketamine/xylazine, as appropriate. Analgesics were provided for procedures expected to cause pain. This included the use of medications (Zoletil 15 mg/kg IP, xylazine 5 mg/kg IP, isoflurane 1–4% inhalation, proparacaine 0.5% ophthalmic solution), the humane termination criteria (20% body weight loss, eye rupture, severe inflammation/edema, etc.). All mice remained in good health without any complications or significant abnormalities throughout the study period.

### 2.14. Establishment of a Mouse Corneal CNV Model and Treatment Conditions

A mouse model of CNV was established in the right eye by placing sutures in the corneal stroma using a microsurgical microscope. Briefly, CNV was induced in the right eye by placing three 10-0 nylon sutures equally spaced in the corneal stromal layer under a surgical microscope. Sutures were inserted approximately <2.0 mm from the limbus and maintained until neovascularization was confirmed. After establishing the model, mice (*n* = 6 per group) were randomly assigned to five groups: Normal control group (no suturing and injection; Normal), a Bevacizumab-treated group (Bevacizumab), a *VEGFA* siRNA-FAM-loaded exosome-treated group (*VEGFA* siRNA-FAM-EXO), a non-loaded Exosome-treated group (Exosome), and a PBS-treated control group (PBS). Intrastromal injections were administered on days 7, 9, 12, 14, and 17, and mice were euthanized for tissue collection on day 21. The treatment group received either *VEGFA* siRNA-FAM-loaded exosomes or exosomes, while the control group received bevacizumab or PBS on the same injection schedule. The normal group was not subjected to corneal suturing or intrastromal injection. Specifically, *VEGFA* siRNA-FAM-loaded exosomes were administered as a 5 µL intrastromal injection, with a dose of 1 × 10^6^ particles per day. In the exosome-only group, an equivalent dose of exosomes (5 µL; 1 × 10^6^ particles/day) was injected using the same method. *VEGFA* siRNA-FAM (total 20 nmol) was purchased from Bioneer Co., Ltd. (Daejeon, Republic of Korea) and stored at −20 °C, protected from light, according to the manufacturer’s instructions.

As an anti-VEGF positive control, bevacizumab (Avastin^®^; Genentech/Roche, South San Francisco, CA, USA) was stored at 2–8 °C, diluted with sterile PBS to a final concentration of 2.5–5 mg/mL immediately before use, and administered using a 30–33G needle by injecting 5–10 μL parallel to the anterior chamber and into the mid-stromal layer near the limbus. For clarity, the bevacizumab control was administered according to the standard anti-VEGF corneal model protocol, while the *VEGFA* siRNA-FAM-loaded exosomes were administered intrastromally at a dose of 5 μL (1 × 10^6^ particles) according to the schedule described above. These two regimens differ in dosing methods (protein mass for bevacizumab and particle number for exosomes), injection volume, and tissue pharmacokinetic properties. Therefore, unless otherwise stated, comparisons of efficacy between the treatments should be interpreted in light of these methodological differences. The PBS group received 5 μL of sterile PBS by the same method. Considering the thin cornea and small ocular structure of the mouse, a fine needle in the 30–33G range was used. This gauge was selected in this study because it is suitable for delivering small amounts (5–10 μL) into the intermediate stromal layer while minimizing tissue damage and drug reflux and ensuring reproducibility of small injections. Group assignment was randomized, and all evaluators were blinded to treatment conditions. At the study endpoint, eyes were enucleated, and treatment efficacy was evaluated by histological (including hematoxylin and eosin (H&E) staining) and immuno-histochemical analyses.

### 2.15. Assessment of Corneal Neovascularization

To evaluate the extent of CNV following treatment with *VEGFA* siRNA-FAM-loaded exosomes, bevacizumab, exosomes, or PBS, serial assessments were performed using an LSM (Carl Zeiss AG, Oberkochen, Germany). Digital images of the cornea were acquired and quantitatively analyzed using ImageJ software (National Institutes of Health). The angiogenesis index was calculated as a standardized composite metric that incorporates both vessel length and vessel number [14]. Vessel number was scored on a four-point scale: 0 indicated no visible vessels; 1 indicated fewer than 10 vessels; 2 indicated 10 or more vessels with the iris still visible; and 3 indicated 10 or more vessels with the iris obscured. Neovascular length was measured in millimeters from the limbus to the tip of the longest vessel. The angiogenesis index was then calculated as vessel length (mm) × the vessel number score.

### 2.16. H&E Staining

Paraffin-embedded ocular tissues were fixed in 10% neutral buffered formalin for 24 to 48 h, dehydrated through a graded ethanol series, cleared in xylene, and embedded in paraffin. Sections 4–5 μm thick were deparaffinized in xylene, rehydrated through decreasing concentrations of ethanol to water, and stained with hematoxylin for 3–5 min. After rinsing, slides were briefly differentiated in 0.3% acidic alcohol, blued with Scott’s tap water substitute, and counterstained with eosin Y for 30–60 s. Following staining, the sections were dehydrated in ethanol, cleared in xylene, and mounted with Canada balsam. Bright-field images were captured using an optical microscope (Olympus Corp., Tokyo, Japan) under identical imaging settings across all experimental groups.

### 2.17. Statistical Analysis

Data were presented as mean ± standard error of the mean (SEM). Normal distribution and homogeneity of variance were confirmed for all groups before analysis. Statistical comparisons across treatment groups and time points were conducted using one-way analysis of variance (ANOVA), followed by Tukey’s multiple comparison test as a post hoc analysis. Statistical significance was defined as * *p* < 0.05, ** *p* < 0.01, and *** *p* < 0.001. No predetermined inclusion or exclusion criteria were applied in this study.

## 3. Results

### 3.1. Structural and Molecular Validation of Purified MSC-Derived Exosomes

**Figure 1:**
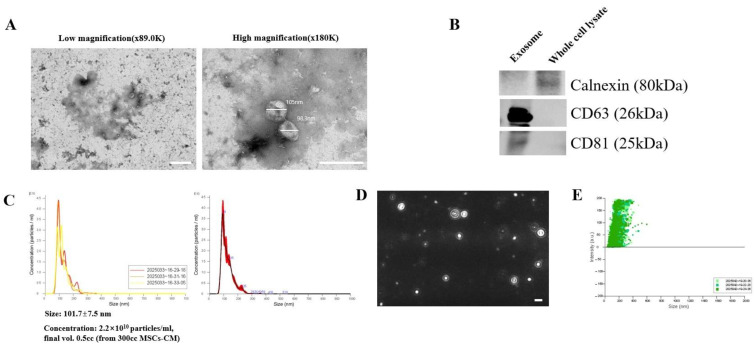
Characterization of exosomes. Exosomes secreted from MSCs were purified using a chromatographic exosome separation method, and their presence was confirmed by (**A**) TEM images, (**B**) Western Blot analysis, (**C**) NTA nanosite analysis, (**D**) NTA tracking image of exosome, and (**E**) exosome intensity/size graph. Bars represent 200 nm.

High-purity exosomes were isolated from MSCs using a chromatography-based method that exploits particle density differences. Their purity and identity were validated through structural and molecular analyses. TEM confirmed the presence of vesicles with characteristic cup-shaped morphology and diameters ranging approximately 40–110 nm (Figure 1A). Vesicles appeared intact, with no evidence of rupture or aggregation. Western Blot detected exosomal markers CD63 and CD81 in the exosome fraction, while calnexin, an endoplasmic reticulum protein, was absent, confirming minimal contamination from intracellular components (Figure 1B).

NTA was used to measure particle size and concentration (Figure 1C–E). Exosomes purified from 300 mL of MSC-conditioned medium had a mean diameter of 101.7 ± 7.5 nm and a concentration of 2.2 × 10^10^ particles/mL (Figure 1C). The NTA tracking image showed real-time Brownian motion of particles, with each exosome appearing as a bright dot and its trajectory marked by a circular path (Figure 1D). The dynamic signal distribution and absence of stationary particles or aggregates suggest minimal nonspecific contamination and support measurement accuracy. The size-intensity plot (Figure 1E) showed positive intensity values, confirming that detected events exceeded the background noise threshold in both scattering and fluorescence channels and likely represent true exosomes or exosome-like nanoparticles.

### 3.2. Enhanced Uptake and Cytoplasmic Release of Exosomal siRNA

To improve both exosome uptake by recipient cells and cytoplasmic release of the delivered siRNA cargo, we optimized transduction conditions using fluorescence-labeled siRNA (Figure S1). A schematic overview of the exosome-based siRNA delivery process is shown in Figure S1A, illustrating key steps including siRNA loading, cellular uptake, cytoplasmic release, and the gradual increase in fluorescence signal over time. In preliminary experiments, exosomes were loaded with Texas Red-labeled siRNA to evaluate transduction efficiency at different time points. Fluorescence microscopy revealed progressively increased red fluorescence with longer exposure durations, with maximal signal observed at 48 h post-transduction (Figure S1B). Based on these results, *VEGFA* siRNA labeled with FAM was used for subsequent experiments.

### 3.3. VEGFA Knockdown and Cellular Effects Induced by VEGFA siRNA-FAM-Loaded Exosomes

**Figure 2:**
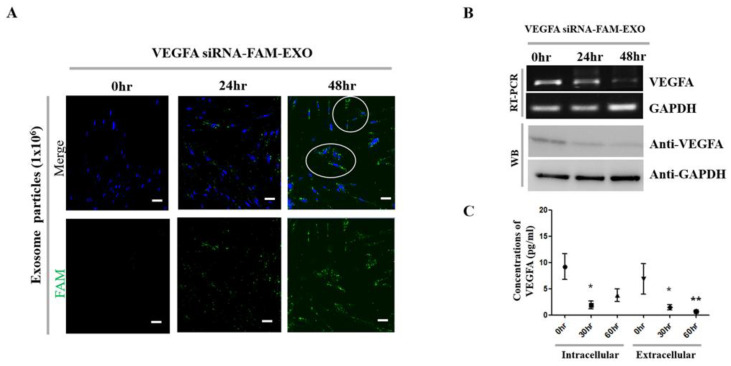
*VEGFA* siRNA-FAM-loaded exosome-mediated introduction into MSCs. (**A**) *VEGFA* siRNA-FAM (green fluorescence) was successfully injected into exosomes and delivered to target cells. White circles represent exosomes fluorescently labeled with *VEGFA* siRNA-FAM. (**B**) RT-PCR and Western Blot analysis of MSCs over time after treatment. A decrease in *VEGFA* mRNA and protein expression was observed under the same conditions. (**C**) Intracellular and extracellular VEGFA levels in MSCs were quantified over time using a multiplex assay. Bars represent 200 μm. * *p* < 0.05 and ** *p* < 0.01 were considered significant differences.

Exosomes loaded with *VEGFA* siRNA-FAM were applied to MSCs to assess intracellular delivery efficiency and gene-silencing effects (Figure 2A). Confocal microscopy revealed cytoplasmic FAM fluorescence (green) as early as 24 h post-treatment, with markedly increased signal intensity at 48 h, indicating efficient intracellular uptake. Morphological changes were also observed at 48 h, including altered nuclear shape and signs of nuclear degradation, which are suggestive of apoptosis. Quantitative RT-PCR analysis showed a gradual decrease in *VEGFA* mRNA expression at 24 and 48 h, whereas GAPDH expression remained unchanged. Western Blot confirmed a corresponding time-dependent decrease in VEGFA protein, with the lowest levels observed at 48 h; GAPDH was used as a loading control (Figure 2B).

To further quantify protein-level suppression, VEGFA was measured in both intracellular and extracellular fractions at 0, 30, and 60 h post-treatment (Figure 2C). Intracellular VEGFA levels decreased to approximately 0.21-fold at 30 h (* *p* < 0.05) and remained suppressed at 0.41-fold at 60 h. Similarly, extracellular VEGFA levels were reduced to 0.32-fold at 30 h (* *p* < 0.05) and 0.49-fold at 60 h (** *p* < 0.01), relative to baseline. These results demonstrate that *VEGFA* siRNA-FAM-loaded exosomes achieved efficient cytoplasmic delivery, induced robust VEGFA knockdown at both transcript and protein levels, and produced consistent reductions in both intracellular and secreted VEGFA protein, accompanied by observable morphological changes.

### 3.4. Time-Dependent Effects of VEGFA siRNA-FAM-Loaded Exosomes on MSC Viability, Apoptosis, and HUVEC Tube Formation

**Figure 3:**
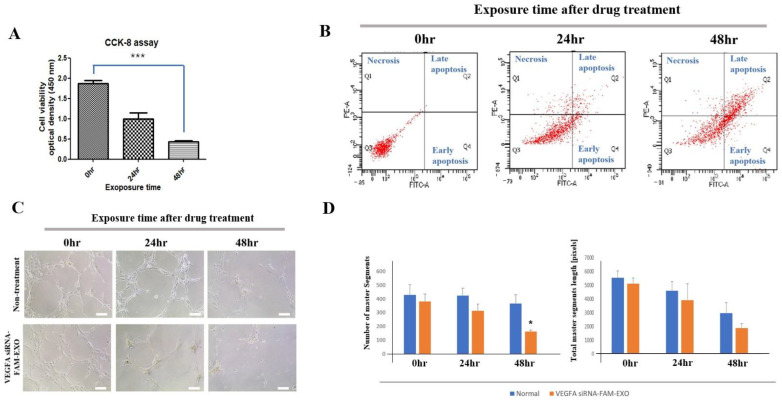
Decreased cell viability, increased apoptosis, and inhibited HUVEC-derived tube formation in MSCs induced by *VEGFA* siRNA-FAM-loaded exosomes in vitro. (**A**) Cell viability (CCK 8) as a function of *VEGFA* siRNA-FAM-loaded exosomes into the target cells according to exposure time. (**B**) Time-dependent effects of *VEGFA* siRNA treatment as determined by FACS analysis. Terms: Q1, necrosis; Q2, late apoptosis; Q3, survival; Q4, early apoptosis. (**C**) Morphological changes in tube formation in HUVECs over time after *VEGFA* siRNA-FAM-loaded exosome treatment. (**D**) Quantitative graph of the number of master segments involved in tube formation and total master segment length. Statistics: * *p* < 0.05 and *** *p* < 0.001 were considered significant differences, and were evaluated by one-way ANOVA followed by Tukey’s multiple comparisons. Scale bar = 200 μm.

MSC viability decreased in a time-dependent manner following treatment with *VEGFA* siRNA-FAM-loaded exosomes. According to CCK-8 assay results, cell viability was significantly reduced to 53% of baseline at 24 h and further declined to 23% at 48 h, with the greatest reduction observed at the 48-h time point (Figure 3A; *n* = 8, *** *p* < 0.001). These results were supported by Annexin V/PI staining and flow cytometric analysis. At 24 h, the proportion of viable cells (Q3) decreased from 100% (baseline) to 84.0 ± 2.51%, while early apoptotic cells (Q4) increased to 8.43 ± 4.27% and late apoptotic cells (Q2) to 2.70 ± 1.15%. At 48 h, viability (Q3) decreased further to 38.37 ± 3.04%, with early apoptosis (Q4) rising to 20.17 ± 3.80% and late apoptosis (Q2) to 36.40 ± 6.54%.

The proportion of necrotic cells (Q1) remained low and relatively unchanged over time, at 5.13 ± 2.15% at 24 h and 5.03 ± 3.91% at 48 h, suggesting apoptosis was the predominant mode of cell death (Figure 3B; *n* = 5). The anti-angiogenic activity of *VEGFA* siRNA-FAM-loaded exosomes was assessed using a HUVEC tube formation assay. At 48 h post-treatment, treated cells exhibited substantial disruption in network formation, characterized by reduced branching and loss of interconnectivity, whereas untreated control cells retained intact vascular structures (Figure 3C). Quantitative image analysis demonstrated a progressive decline in both the number of master segments and total branch length from 24 to 48 h. Master segments decreased from 0.82-fold to 0.42-fold, and total branch length from 0.76-fold to 0.36-fold, relative to control (Figure 3D; *n* = 5). Notably, the number of master segments at 48 h was significantly lower than that of the control group (* *p* < 0.05; Figure 3D).

### 3.5. Body Weight and Health Status of Mice

During the experimental period, body weight changes of BALB/c mice were monitored regularly. As shown in Figure S2, all groups including Normal, Bevacizumab, *VEGFA* siRNA-FAM-EXO, Exosome, and PBS exhibited gradual increases in body weight over time. No significant signs of adverse health effects were observed, and all mice remained healthy throughout the study period.

### 3.6. In Vivo Delivery and Stromal Uptake of VEGFA siRNA-FAM-Loaded Exosomes

**Figure 4:**
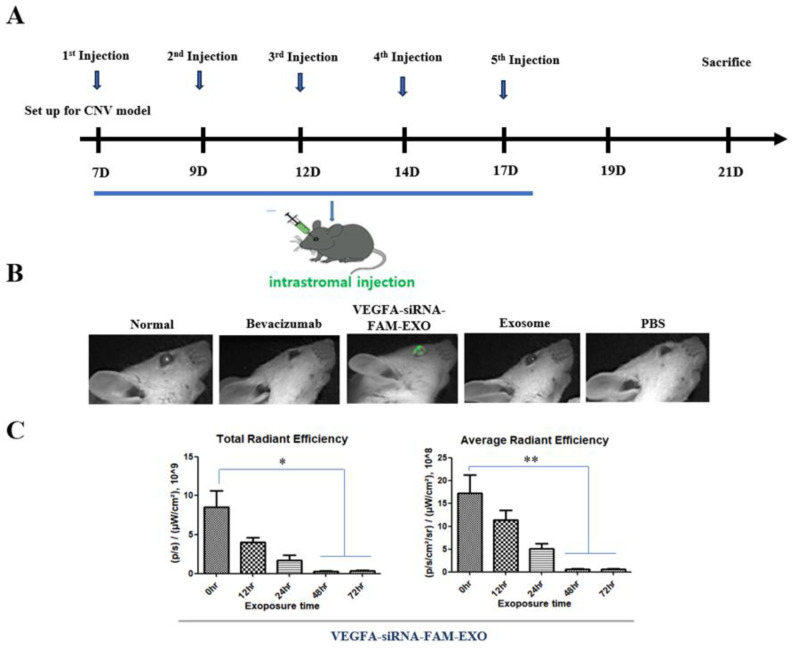
Establishment of conditions for the treatment of *VEGFA* siRNA-FAM-loaded exosomes in the right eye of each mouse with the CNV model in vivo. (**A**) Experimental timeline. After successful establishment of the CNV model, intrastromal injections were performed into the corneal stroma on days 7, 9, 12, 14, and 17, and sacrifice and biopsy were performed on day 21. (**B**) Fluorescence imaging after treatment with *VEGFA* siRNA-FAM-loaded exosomes. Fluorescence images of the *VEGFA* siRNA-FAM-EXO-treated group are shown. Green signal (FAM) indicates that *VEGFA* siRNA-loaded exosomes have successfully entered the corneal stroma. (**C**) Quantification of total radiative efficiency (TRE) and average radiative efficiency (AER) at 0, 12, 24, 48, and 72 h after *VEGFA* siRNA-FAM-EXO treatment. Data are presented as mean ± standard error. Statistical analysis was performed using one-way ANOVA followed by Tukey’s multiple comparison test. Standard criteria: * *p* < 0.05, ** *p* < 0.01.

To assess in vivo delivery and corneal stromal uptake of *VEGFA* siRNA-FAM-loaded exosomes, intrastromal injections were performed on days 7, 9, 12, 14, and 17 following CNV induction in mice. Animals were sacrificed on day 21 for tissue collection and analysis (Figure 4A). Fluorescence imaging revealed distinct green FAM signals in the corneal stroma of the *VEGFA* siRNA-FAM-loaded exosomes (*VEGFA* siRNA-FAM–EXO) group, confirming successful delivery and tissue uptake. In contrast, only background-level fluorescence was detected in the Normal, Exosome-only (Exosome), and PBS groups, and no FAM signal was observed in the Bevacizumab group (Figure 4B). Total radiative efficiency represents the cumulative fluorescence signal across the defined region of interest. Quantitative analysis showed a time-dependent decrease in total radiative efficiency, which declined from 8.50 at 0 h to 4.04 at 12 h, 1.72 at 24 h, and reached 0.27 at 48 h, remaining low at 0.32 at 72 h. Notably, total radiative efficiency was significantly reduced at 48 and 72 h compared to baseline (0 h) (Figure 4C; *n* = 6, * *p* < 0.05). Similarly, average radiative efficiency, defined as fluorescence intensity normalized to the area of the region of interest, decreased from 17.64 at 0 h to 11.37 at 12 h, 5.05 at 24 h, and dropped sharply to 0.61 at 48 h, remaining stable at 0.60 at 72 h. The reductions at 48 and 72 h were statistically significant compared to 0 h (Figure 4C; *n* = 6, ** *p* < 0.01). These data confirm that *VEGFA* siRNA-FAM-loaded exosomes were effectively delivered and retained within the corneal stroma, with peak signal intensity observed at early time points and a rapid decline between 24 and 48 h, suggesting intracellular uptake or signal clearance.

### 3.7. In Vivo Inhibition of CNV by VEGFA siRNA–FAM-Loaded Exosomes

**Figure 5:**
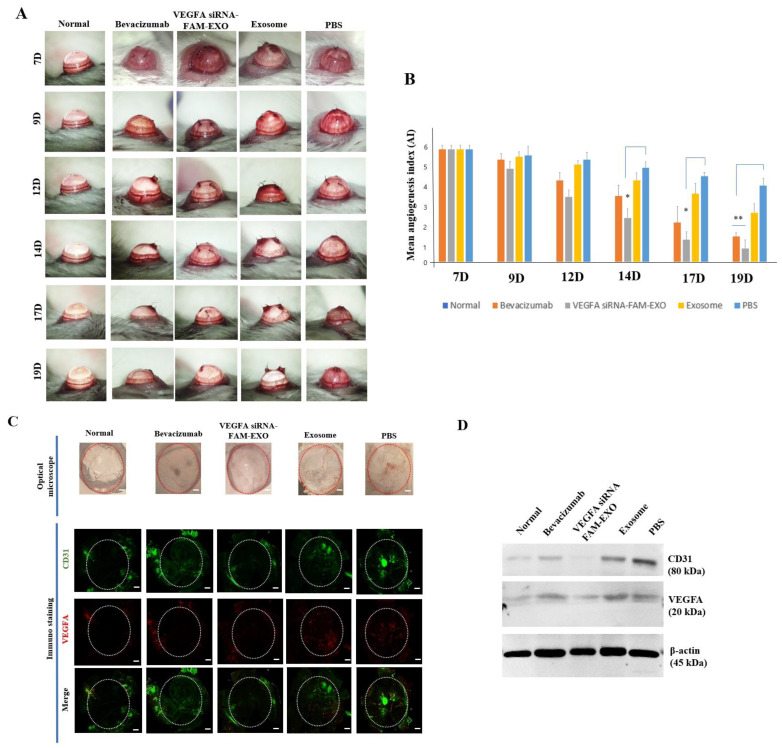
*VEGFA* siRNA-FAM-loaded exosomes inhibit corneal neovascularization in vivo. (**A**) Representative anterior segment images from each group (Normal, Bevacizumab, *VEGFA* siRNA-FAM-EXO, Exosomes, PBS). Images were taken on days 7, 9, 12, 14, 17, and 19 after model induction to compare changes in neovascularization over time. (**B**) Quantitative results of the angiogenic index (AI) (mean ± standard error, *n* = 6). A significant decrease was observed in the *VEGFA* siRNA-FAM-EXO group from days 14 to 19. (**C**) Optical photograph (top) and immunohistochemical staining (bottom) of mouse corneas. The expression levels of CD31 and VEGFA were indicated by their respective fluorescence (green and red) within circles (corneas), and vascular signals were confirmed in the expanded image. (**D**) Western Blot analysis. The expression of CD31 (a marker of vascular endothelial cells) and VEGFA was compared in lysates collected from corneal tissues at day 21 (the time of sacrifice) in five groups. * *p* < 0.05 and ** *p* < 0.01 were considered significant differences. Scale bar = 200 μm.

During the early phase of CNV induction (Days 7–12), anterior segment examination showed no significant differences between the *VEGFA* siRNA-FAM–EXO group and the other groups. However, by Day 14, a clear reduction in the extent and density of neovascularization moving from the corneal periphery toward the center was observed in the *VEGFA* siRNA-FAM-EXO-treated group (Figure 5A). In contrast, on Day 14, the Bevacizumab group exhibited only mild inhibition, while both the Exosome and PBS groups continued to display progressive vascular growth. Quantitative analysis of the angiogenesis index confirmed a significant inhibitory effect in the *VEGFA* siRNA-FAM-EXO-treated group beginning on Day 14, with the maximum suppression observed between Days 17 and 19. Compared to Day 7, the angiogenesis index decreased by 0.92-fold on Day 9, 0.83-fold on Day 12, 0.37-fold on Day 14, 0.30-fold on Day 17, and 0.12-fold on Day 19 (Figure 5B; mean ± SE, *n* = 6; * *p* < 0.05 on Days 14 and 17; ** *p* < 0.01 on Day 19). The decrease was significantly greater in the *VEGFA* siRNA-FAM-EXO-treated group compared to the Exosome and PBS control groups.

Immunofluorescence analysis further confirmed these results. CD31 (green) and VEGFA (red) expression in the cornea was significantly decreased in the *VEGFA* siRNA-FAM-EXO-treated group, showing a clear gradient of decreasing fluorescence intensity from the peripheral to the central cornea (Figure 5C). The Bevacizumab group exhibited moderate reductions, while strong signals persisted in the Exosome and PBS groups. As expected, the Normal control group showed no detectable signal. Western Blot analysis corroborated these results at the protein level. The *VEGFA* siRNA-FAM-EXO-treated group showed the lowest expression of CD31 (80 kDa) and VEGFA (20 kDa), followed by intermediate levels in the Bevacizumab group, with high levels observed in the Exosome and PBS groups (Figure 5D).

### 3.8. Histological Suppression of CNV and Inflammation, and Systemic Safety of VEGFA siRNA-FAM-Loaded Exosomes

**Figure 6:**
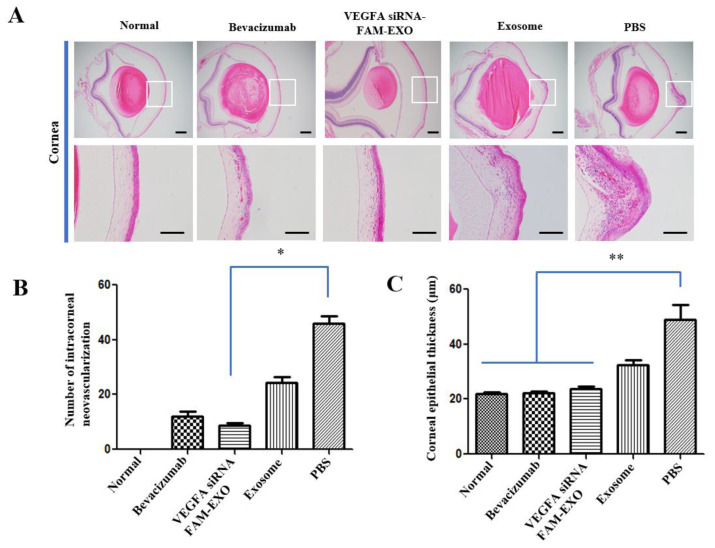
*VEGFA* siRNA-FAM-loaded exosomes reduce angiogenesis and attenuate inflammatory changes. (**A**) H&E-stained mouse corneal tissue images. Anterior segment sections (top) and enlarged corneal sections (bottom) are shown for each group: Normal, Bevacizumab, *VEGFA* siRNA-FAM-EXO, Exosome, and PBS. Neovascular invasion and inflammatory hypertrophy were attenuated in the *VEGFA* siRNA-FAM-EXO group. Scale bar = 200 μm. (**B**) Quantification of the number of corneal neovascularization (mean ± standard error, *n* = 4). A significant reduction in blood vessels was observed in the *VEGFA* siRNA-FAM-EXO group compared to PBS. (**C**) Quantification of corneal epithelial thickness (mean ± standard error, *n* = 6). Inflammatory epithelial hyperplasia was suppressed in the *VEGFA* siRNA-FAM-EXO group. Statistical analysis was performed using one-way ANOVA followed by Tukey’s multiple comparisons, and the significance levels were * *p* < 0.05 and ** *p* < 0.01.

H&E staining revealed a significant reduction in peripheral vascular infiltration and epithelial thickening in the *VEGFA* siRNA-FAM-EXO-treated group (Figure 6A). Both low- and high-magnification images showed decreased stromal neovascularization and inflammatory cell infiltration, along with a uniformly thin epithelial layer. The Bevacizumab group exhibited similar but less pronounced suppression of neovascularization and inflammation. Conversely, the Exosome and PBS groups showed prominent stromal neovascularization, dense inflammatory infiltration, and increased epithelial thickness. The Normal control group exhibited no pathological changes. Further analysis of inflammatory cell types revealed high densities of multinucleated neutrophils and histiocytes/macrophages, along with localized lymphocytes and plasma cells in the Exo-some-only and PBS groups.

In contrast, the *VEGFA* siRNA-FAM-EXO-treated group had significantly fewer neutrophils and macrophages, with minimal lymphocyte infiltration, suggesting reduced immune cell recruitment. Quantitative analysis supported these histological findings. The number of stromal neovessels in the *VEGFA* siRNA-FAM-EXO-treated group was significantly lower than in the PBS group, reduced to 0.19-fold (8.83 ± 1.38 vs. 46.67 ± 2.32; *n* = 6, * *p* < 0.05) (Figure 6B). The Bevacizumab group showed a similar reduction to 0.22-fold (10.17 ± 1.33), while the Exosome group maintained 0.52-fold (24.33 ± 1.71). No neovascularization was observed in the Normal group (0.00 ± 0.00). Corneal epithelial thickness was also significantly reduced. The *VEGFA* siRNA-FAM-EXO-treated group exhibited the thinnest and most uniform epithelium, measuring 23.44 ± 0.69 μm, representing a 0.45-fold decrease compared to the PBS group (49.20 ± 3.99 μm; ** *p* < 0.01) (Figure 6C).

The Bevacizumab group measured 22.39 ± 0.31 μm (0.43-fold; ** *p* < 0.01), and the Exosome group measured 32.47 ± 1.22 μm (0.62-fold). The Normal group also showed significantly thinner epithelium than PBS (21.67 ± 0.45 μm; ** *p* < 0.01). Systemic safety was assessed through histopathological and macroscopic evaluations. H&E-stained sections of the liver, kidney, and spleen revealed no abnormalities such as inflammation, necrosis, hemorrhage, or fibrosis in any group (Figure S3A). Macroscopic examination at necropsy showed no signs of discoloration, hypertrophy, atrophy, or hemorrhage (Figure S3B). Additionally, organ weight analysis indicated no significant differences among groups for the liver, kidney, or spleen (Figure S3C).

### 3.9. VEGFA siRNA-FAM-Loaded Exosomes Inhibit PI3K/Akt Signaling and Induce Apoptosis In Vivo

**Figure 7:**
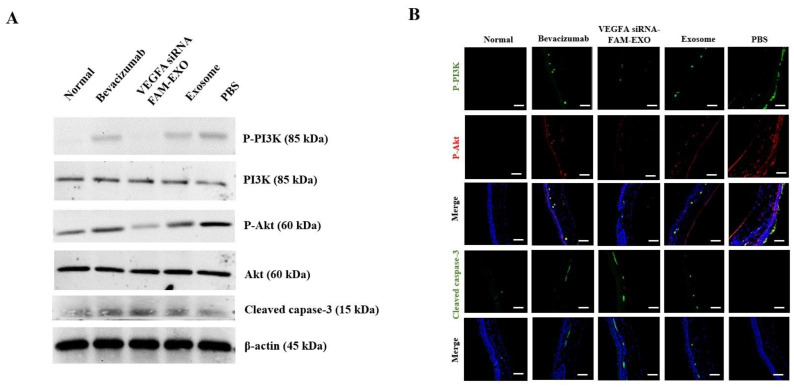
*VEGFA* siRNA-FAM-loaded exosomes inhibit PI3K/Akt signaling and induce apoptosis. (**A**) Western Blot analysis showed that the *VEGFA* siRNA-FAM-EXO group showed decreased levels of phosphorylated PI3K (p-PI3K, 85 kDa) and phosphorylated Akt (p-Akt, 60 kDa), while cleaved caspase 3 (15–17 kDa) increased. Total PI3K (85 kDa) and total Akt (60 kDa) were presented as comparative indices, and β-actin (45 kDa) was used as a loading control. Band intensities were quantified by normalization to β-actin. (**B**) Immunofluorescence staining analysis. The expression of p-PI3K (green), p-Akt (red), and cleaved caspase-3 (green) in each treatment group (Normal, Bevacizumab, *VEGFA* siRNA-FAM-EXO, Exosome, and PBS) was presented as a merged image with DAPI (blue) nuclear staining. The *VEGFA* siRNA-FAM-EXO group showed a decrease in p-PI3K and p-Akt signals and an increase in cleaved caspase-3 signals, consistent with the Western Blot results. The white scale bar represents 200 μm.

To investigate the mechanism underlying the therapeutic effects of *VEGFA* siRNA-FAM-loaded exosomes, molecular and histological analyses were conducted in the CNV mouse model. These exosomes induced apoptosis in neovascularized corneal tissue by downregulating the phosphoinositide 3-kinase (PI3K)/Akt signaling pathway and upregulating cleaved caspase-3 expression, thereby contributing to CNV suppression (Figure 7A,B). Western Blot analysis of corneal tissue revealed a significant decrease in phosphorylated PI3K (p-PI3K, 85 kDa) and phosphorylated Akt (p-Akt, 60 kDa) in the *VEGFA* siRNA-FAM-EXO group (Figure 7A). In contrast, total PI3K (85 kDa) and total Akt (60 kDa) levels remained unchanged across all groups, with β-actin (45 kDa) serving as the loading control. Importantly, cleaved caspase-3 (15–17 kDa), a key marker of apoptosis, was significantly elevated in the *VEGFA* siRNA-FAM-EXO-treated group, indicating activation of apoptotic signaling following inhibition of the PI3K/Akt pathway.

The Bevacizumab group exhibited a moderate decrease in p-PI3K and p-Akt and a slight increase in cleaved caspase-3. The Exosome and PBS groups maintained elevated p-PI3K and p-Akt expression and showed low levels of cleaved caspase-3, consistent with limited apoptotic activity. These molecular findings were corroborated by immunofluorescence staining (Figure 7B). In the *VEGFA* siRNA-FAM-EXO-treated group, corneal tissue displayed reduced fluorescence signals for p-PI3K (green) and p-Akt (red), and enhanced signal for cleaved caspase-3 (green). These markers were clearly localized within the corneal region and visualized in merged images with DAPI-stained nuclei (blue). The Bevacizumab group demonstrated partial suppression of p-PI3K/p-Akt and a moderate increase in cleaved caspase-3. In contrast, the Exosome and PBS groups exhibited strong p-PI3K and p-Akt signals and weak cleaved caspase-3 expression. Treatment with *VEGFA* siRNA loaded into exosomes was associated with decreased PI3K/Akt phosphorylation and increased cleaved caspase-3, consistent with reduced endothelial survival signaling. These results indicate a correlation between VEGFA suppression and activation of apoptotic pathways.

## 4. Discussion

Standard anti-VEGF antibody therapy suppresses angiogenesis by binding to and neutralizing extracellular VEGF. However, its clinical utility is limited by a short intraocular half-life, the need for frequent administration, and variable patient responses due to intrinsic or acquired resistance mechanisms [3,4]. In contrast, the exosome-based siRNA approach presented in this study targets VEGF expression upstream at the mRNA level, disrupting survival signaling more fundamentally. This mechanism offers the potential for more sustained inhibition of angiogenesis and reduced dosing frequency [10,11]. Additionally, exosomes provide stable encapsulation and delivery of siRNA, further supporting extended therapeutic efficacy [10,11]. We validated the purity and quality of MSC-derived exosomes through orthogonal structural and molecular characterization (Figure 1A–E) [15,16]. These exosomes demonstrated time-dependent uptake, cytosolic release, and RISC-mediated gene silencing (Figure 2A–C and Figure 3A–D) [17,18]. Mechanistically, the anti-angiogenic effects were driven by the inhibition of the PI3K–Akt pathway, suppression of caspase–3–mediated apoptosis (Figure 2, Figure 3, Figure 5 and Figure 7) [19,20,21].

In vivo, repeated intrastromal injections resulted in the rapid and sustained localization of exosomes within the corneal stroma (Figure 4A–C) [17,22,23]. In this exploratory preclinical study, a single exosome dose of 1 × 10^6^ particles per administration was selected based on prior literature and our preliminary data demonstrating effective anti-angiogenic activity with acceptable tolerability [24,25]. This dose selection also conformed to the 3R ethical principles by minimizing animal use while clearly establishing proof-of-concept [26]. Throughout the study, the health status of the BALB/c mice was closely monitored, with consistent body weight gain observed (Figure S2) and no significant clinical abnormalities or adverse effects detected. Humane endpoints, including criteria such as 20% body weight loss and severe ocular inflammation, were strictly adhered to in accordance with institutional ethical guidelines, ensuring the welfare of the animals. Notably, the anesthetic agents Zoletil and xylazine were administered via intraperitoneal injection (IP) rather than intramuscular (IM) injection. This modification was made to minimize procedural pain and distress in rodents, thereby enhancing animal welfare without compromising the reliability of experimental outcomes. This adherence to refined animal care protocols underscores our commitment to ethical research practices and contributes to the reproducibility and validity of the study findings. 

The results of this study suggest that intrastromal delivery of MSC-derived exosomes can effectively reduce pathological neovascularization by suppressing *VEGFA* mRNA levels. This is consistent with recent reports showing that the route of administration significantly influences therapeutic response. For example, Soleimani et al. compared intrastromal and subconjunctival delivery of MSCs and reported differences in tissue distribution and therapeutic efficacy depending on the route of administration. In particular, they suggested that local intrastromal delivery was advantageous in maintaining drug concentrations at the target site [27]. Therefore, the decision to adopt intrastromal exosome delivery in this study was based on these mechanistic and pharmacodynamic advantages. Neovascular regression became evident by Day 14, with peak inhibition between Days 17 and 19 (Figure 5A,B) [19,20,28]. This time-dependent effect was accompanied by a reduction in CD31 and VEGFA expression (immunofluorescence and Western Blot), with a gradient of signal loss from the limbus to the central cornea (Figure 5C,D), supporting efficient axial targeting. Suppression of PI3K and Akt phosphorylation, alongside elevated cleaved caspase-3 levels (Figure 7A,B) [21,29], further corroborated the therapeutic mechanism. The preclinical importance of this study is highlighted by the multifaceted suppression of pathological CNV. First, the cornea maintains transparency and refractive homogeneity due to its avascularity. Reducing pathological neovascularization is therefore likely to improve visual acuity and contrast sensitivity by decreasing optical scatter and astigmatic distortion [30,31,32]. Second, *VEGFA* siRNA-FAM-loaded exosomes, acting as anti-angiogenic agents, significantly attenuate neovascular growth, thereby preventing the accelerated development of corneal opacity and scar formation caused by lipid and protein deposition and fibrosis. The observed reduction in the angiogenesis index, along with sustained decreases in CD31 and VEGFA expression, suggests slowed disease progression and the creation of a tissue environment favorable for restoring and preserving corneal clarity (Figure 5A–D) [19,20,32,33].

Third, VEGFA axis inhibition also reduces the inflammatory components of CNV. The concurrent decrease in inflammatory cell infiltration, including neutrophils, macrophages, and lymphocytes, as well as the normalization of epithelial thickness, suggests that suppression of VEGFA disrupts the vascular-inflammatory cycle. This may lower the risk of recurrence, pain, and epithelial instability (Figure 6A–C) [34,35]. Fourth, from an immunological perspective, reduced vascular density decreases the likelihood of graft rejection and immune complications, potentially enhancing the success of pre- and post-keratoplasty interventions [31,32]. Fifth, key limitations of current anti-VEGF protein therapies, including repeated dosing requirements, diffusion barriers, and inter-individual variability, may be overcome by the intracellular targeting and enhanced tissue retention capabilities of exosomal siRNA. These properties support the development of low-frequency, personalized treatment regimens that optimize therapeutic outcomes (Figure 4 and Figure 5) [28,36,37].

At the cellular level, the mechanistic basis for suppressing pathological neovascularization is well understood. Inhibition of VEGFA expression using *VEGFA* siRNA has been observed to induce downregulation of the PI3K–Akt signaling pathway and activation of Caspase-3, leading to apoptosis and functional decline in MSCs and human vascular endothelial cells (HUVECs). This is interpreted as part of the key mechanism underlying the therapeutic effect by inhibiting the survival of cells involved in angiogenesis [38,39,40]. Attenuation of the PI3K–Akt signaling pathway responses downstream of VEGF–VEGFR2 activation impairs endothelial cell survival, migration, and branching (Figure 7) [19,20,21,29]. This is demonstrated by the breakdown of the HUVEC tube network following treatment, characterized by a loss of branching and interconnectivity (Figure 3C) [19,20,33]. While our data show a consistent association between VEGFA silencing, reduced PI3K–Akt signaling, and increased cleaved caspase-3, these observations demonstrate correlation rather than definitive causation. Future pathway modulation experiments (e.g., PI3K/Akt inhibitors or activators, caspase inhibition, and rescue assays) are required to confirm direct mechanistic links. Meanwhile, reductions in both autocrine and paracrine VEGFA levels reduce pro-angiogenic signals in the surrounding microenvironment, thereby alleviating revascularization pressure (Figure 2C; extracellular analysis of conditioned media) [18,24]. Histologically, the decrease in neovascular vessels and the restoration of epithelial thickness uniformity are significant, as they underpin improved visual axis quality and surface stability (Figure 6A–C) [32]. The absence of pathology in the liver, kidney, and spleen, along with stable organ weights in the safety assessment, supports the potential systemic safety of localized therapy (Figure S3A–C) [22,23,41]. As a delivery platform, exosomes offer intrinsic advantages, including low immunogenicity, efficient uptake, and RNA cargo stability [17,18,24]. Coupled with scalable production and rigorous quality control, these features make exosomes promising vehicles for clinical RNA therapeutics [42,43].

Despite these strengths, several limitations need to be addressed. First, there is no direct evidence of RISC engagement. Future studies should use AGO2 immunoprecipitation–qPCR (AGO2-IP qPCR) to confirm siRNA loading into silencing complexes (Figure 2 and Figure 4) [18,24]. Second, strategies to enhance endosomal escape should be explored to improve cytosolic delivery efficiency (Figure 5) [21,27,29]. Third, to better mimic clinical scenarios, efficacy should be validated in medium- to large-scale animal models, while immunogenicity and safety assessments should be conducted according to recommendations for extracellular vesicle therapeutics (Figure 6 and Figure 7) [41]. Fourth, achieving optimal therapeutic outcomes, including sustained corneal clarity and immune tolerance, necessitates comprehensive long-term data encompassing immunogenicity and safety. Such rigorous evaluation guides effective repeat dosing strategies and leverages advancements in exosome engineering and delivery mechanisms [44,45,46,47]. Fifth, *VEGFA* siRNA-exosomes significantly reduced CNV indices to a greater extent. However, since the intraocular pharmacokinetics of anti-VEGF proteins (e.g., half-life of intravitreal bevacizumab) and the local exposure characteristics of exosomes may differ, we plan to verify superiority through PK/PD matching comparison based on equivalent exposure [48,49]. Sixth, the relatively small sample sizes used in some of the experiments in this study may be a limitation that requires careful interpretation of the generalizability and statistical power of the results. Finally, increased cleaved caspase-3 levels confirm apoptosis involvement. Detection of the 15–17 kDa cleaved caspase-3 band supports endothelial apoptosis as a therapeutic endpoint (Figure 2, Figure 3 and Figure 7). However, because these findings are correlational, additional pathway modulation experiments (e.g., inhibitors or structural analysis) are needed to verify causality and strengthen the mechanistic argument (Figure 2, Figure 4, Figure 5, Figure 6 and Figure 7) [21,24,29].

## 5. Conclusions

This study demonstrated a multi-step therapeutic mechanism in which high-quality MSC-derived exosomes enabled efficient intracellular delivery and release of *VEGFA* siRNA, resulting in targeted VEGFA knockdown, suppression of the PI3K–Akt signaling pathway, and activation of caspase-3–mediated apoptosis. These molecular events led to coordinated inhibition of pathological angiogenesis and inflammation, as well as improved functional outcomes, including a significantly reduced angiogenesis index in a mouse model of CNV. The clinical significance of suppressing pathological CNV was emphasized by its potential to preserve visual acuity, lower the risk of recurrence and corneal scarring, improve transplant success rates, and decrease treatment frequency and burden. These findings support exosome-based siRNA therapy as a practical and mechanistically sound alternative to traditional anti-VEGF strategies. By targeting upstream VEGFA expression, this approach overcomes key limitations of current protein-based therapies and provides a solid foundation for further translational and clinical research in ocular vascular diseases.

## Figures and Tables

**Table 1:** *VEGFA* siRNA base sequence and *VEGFA* primer information.

**siRNA**	**Sequences (5′-3′)**	
*VEGFA* siRNA-FAM	Sense strand: FAM-CUG AUA CAG AAC GAU CGA U=tt	
	Antisense strand: AUC GAU CGU UCU GUA UCA G=tt	
**Primer**	**Sequences (5′-3′)**	**Product Size**
*VEGFA*	Sense strand: GATTCAGTTCGAGGAAAGGGAAAGG	160 bp
	Antisense strand: GCATCAACGCGAGTCTGTGTTTTTG	

**Table 2:** Antibody information for Western Blot (in vitro, in vivo) and immunofluorescence staining (in vivo).

**No.**	**Target (Antibody) for Western Blot**	**Application**	**Dilution**	**Supplier (Official Name; Headquarters City/State/ZIP/Country)**
1	p-PI3K	Primary	1:1000	Santa Cruz Biotechnology, Inc.; Santa Cruz, CA, USA
2	PI3K	Primary	1:1000	Santa Cruz Biotechnology, Inc.
3	p-Akt	Primary	1:1000	Cell Signaling Technology, Inc.; Danvers, MA, USA
4	Akt	Primary	1:1000	Cell Signaling Technology, Inc.
5	Cleaved caspase-3	Primary	1:1000	Cell Signaling Technology, Inc.
6	VEGFA	Primary	1:1000	OriGene Technologies, Inc.; Rockville, MD, USA
7	GAPDH (loading control)	Primary	1:2000	Abcam Limited.; Cambridge, UK
8	CD31 (PECAM)	Primary	1:1000	Thermo Fisher Scientific Inc.; Waltham, MA, USA
9	β-actin (loading control)	Primary	1:2000	Santa Cruz Biotechnology, Inc.
10	CD63 (exosome marker, positive)	Primary	1:1000	Abcam Limited.
11	CD81 (exosome marker, positive)	Primary	1:1000	Abcam Limited.
12	Calnexin (exosome marker, negative)	Primary	1:1000	Abcam Limited.
13	peroxidase-labeled anti-rabbit secondary antibodies	Secondary	1:2000	Abcam Limited.
14	peroxidase-labeled anti-mouse secondary antibodies	Secondary	1:2000	Abcam Limited.
**No.**	**Target (Antibody) for** **Immunofluorescence Staining**	**Application**	**Dilution**	**Supplier (Official Name; Headquarters City/State/ZIP/Country)**
1	p-PI3K	Primary	1:100	Santa Cruz Biotechnology, Inc.
2	p-Akt	Primary	1:100	Cell Signaling Technology, Inc.
3	Cleaved caspase-3	Primary	1:100	Cell Signaling Technology, Inc.
4	VEGFA	Primary	1:500	OriGene Technologies, Inc.
5	CD31 (PECAM)	Primary	1:500	Thermo Fisher Scientific Inc.
6	anti-rabbit-Cy2	Secondary	1:500	Santa Cruz Biotechnology, Inc.
7	anti-mouse Alexa 488	Secondary	1:500	Abcam Limited.
8	anti-rabbit Alexa 488	Secondary	1:500	Abcam Limited.
9	DAPI	Nuclear staining	1:1000	Thermo Fisher Scientific Inc.

## Data Availability

All datasets used and/or analyzed during the current study are available from the corresponding author on reasonable request.

## Supplementary Materials

The following supporting information can be downloaded at https://www.mdpi.com/article/10.3390/biomedicines14010246/s1. Figure S1: Establishment of *VEGFA* siRNA-FAM loading conditions into exosomes, confirming high loading efficiency and cellular uptake via confocal microscopy; Figure S2: Time course of body weight changes in BALB/c mice during the experimental period; Figure S3: Systemic safety evaluation of *VEGFA* siRNA-FAM-loaded exosomes in mice, showing no significant pathological findings in organs, macroscopic abnormalities, or changes in organ weights across treatment groups.

## Abbreviations

The following abbreviations are used in this manuscript:

AGO2	Argonaute-2
Akt	Protein kinase B
AMT	Advanced Microscopy Techniques
ANOVA	Analysis of variance
ATCC	American Type Culture Collection
BCA	Bicinchoninic acid
BSA	Bovine serum albumin
CCK-8	Cell Counting Kit-8
cDNA	Complementary DNA
CD31/CD63/CD81	Cluster of Differentiation 31/63/81
CCD	Charge-coupled device
CO_2_	Carbon dioxide
CNV	Corneal neovascularization
DAPI	4′,6-diamidino-2-phenylindole
DMEM	Dulbecco’s Modified Eagle Medium
DNA	Deoxyribonucleic acid
ECL	Enhanced chemiluminescence
EDTA	Ethylenediaminetetraacetic acid
Exo-Fect	Exosome transfection reagent
FACS	Fluorescence-Activated Cell Sorting
FAM	Fluorescein amidite
FBS	Fetal bovine serum
FITC	Fluorescein isothiocyanate
H&E	Hematoxylin and eosin
HUVECs	Human umbilical vein endothelial cells
IACUC	Institutional Animal Care and Use Committee
IgG	Immunoglobulin G
kDa	Kilodalton
LN_2_	Liquid nitrogen
LSGS	Low-serum growth supplement
LSM	Laser scanning microscope
mRNA	Messenger RNA
MSC/MSCs	Mesenchymal stem cell (s)
Nrf2	Nuclear factor erythroid 2–related factor 2
NTA	Nanoparticle tracking analysis
PBS	Phosphate-buffered saline
PE	Phycoerythrin
PI	Propidium iodide
PI3K	Phosphoinositide 3-kinase
PVDF	Polyvinylidene difluoride
qPCR	Quantitative polymerase chain reaction
RIPA	Radioimmunoprecipitation assay
RISC	RNA-induced silencing complex
RNA	Ribonucleic acid
Rpm	Revolutions per minute
RT-PCR	Reverse transcription polymerase chain reaction
SDS	Sodium dodecyl sulfate
SEM	Standard error of the mean
SE	Standard error
siRNA	Small interfering RNA
TEM	Transmission electron microscopy
TBST	Tris-buffered saline with Tween-20
VEGF	Vascular endothelial growth factor
VEGFA	Vascular endothelial growth factor A
VEGFR2	Vascular endothelial growth factor receptor 2
xMAP	Multi-Analyte Profiling (Luminex technology)
