# Supplementary material for: Therapeutic Exosomes Carrying VEGFA siRNA Inhibit Pathological Corneal Angiogenesis via PI3K–Akt–Caspase-3 Signaling
Source: Biomedicines. 2026 Jan 21;14(1):246. doi: 10.3390/biomedicines14010246 (PMC12839048; doi:10.3390/biomedicines14010246)
Supplement: Supplementary file 1 [file biomedicines-14-00246-s001.zip › biomedicines-4087213-supplementary.pdf]

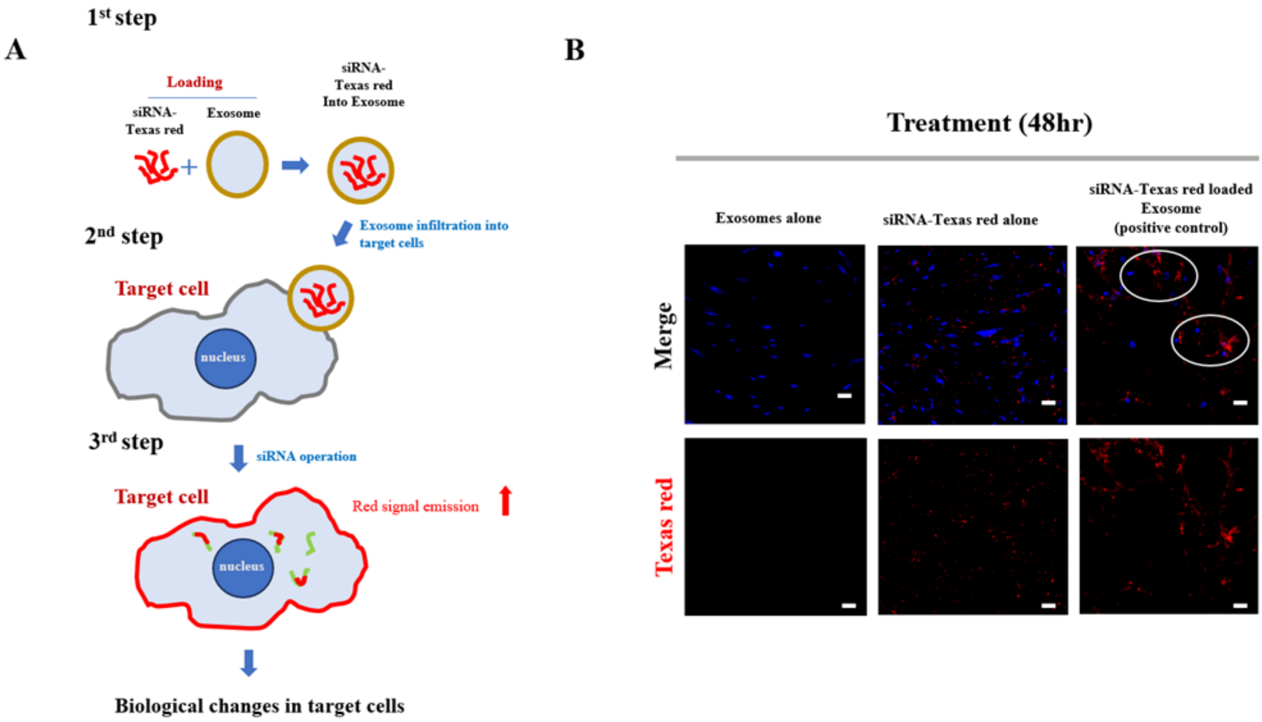


**Figure S1.** To load VEGFA siRNA-FAM into exosomes, we first established the loading conditions using siRNA-TX Red. (**A**) Experimental schematic. (**B**) Positive control results confirming high loading efficiency by directly transfecting purely isolated exosomes with siRNA (TX Red). Confocal microscopy 48 hours after treatment revealed that the area marked by the white circle indicated the cytoplasm of the target cell where the siRNA-TX-loaded exosomes emitted a red signal. The white bar indicates 200 μm.

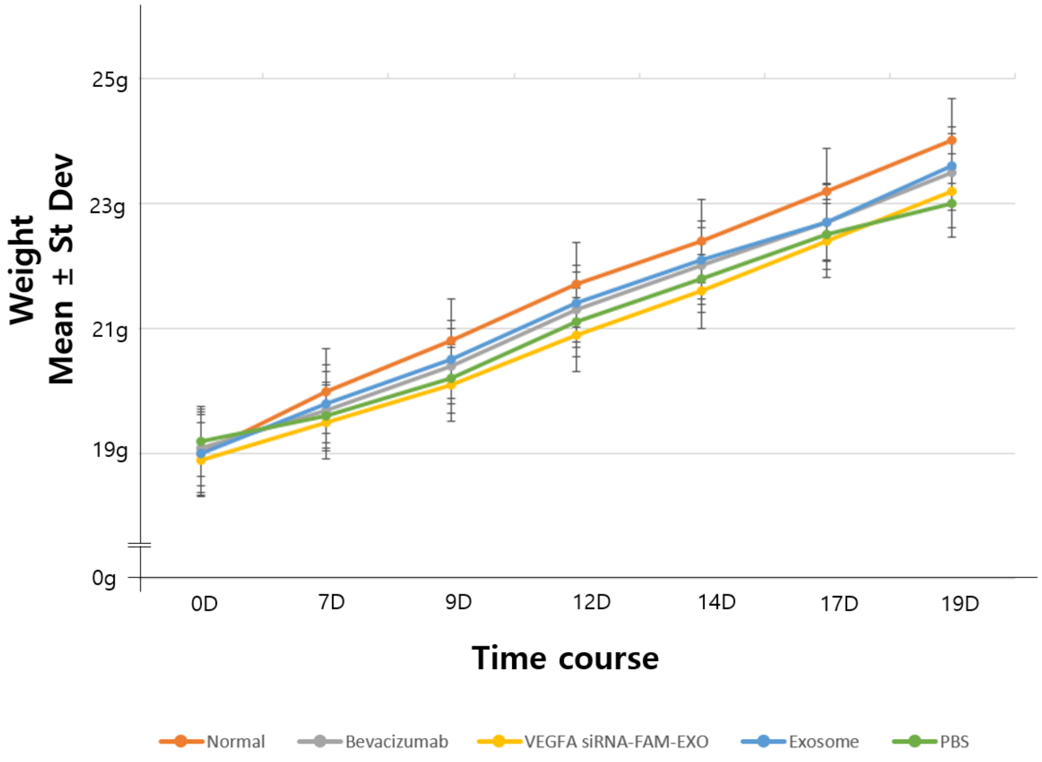

**Figure S2**. Time course of body weight changes in BALB/c mice during the experimental period. Body weights (mean ± standard deviation) were measured at indicated days from day 0 to day 19 in five groups: Normal (orange), Bevacizumab (gray), VEGFA siRNA-FAM-EXO (yellow), Exosome (blue), and PBS (green). All groups showed a gradual increase in body weight with no significant signs of adverse health effects.

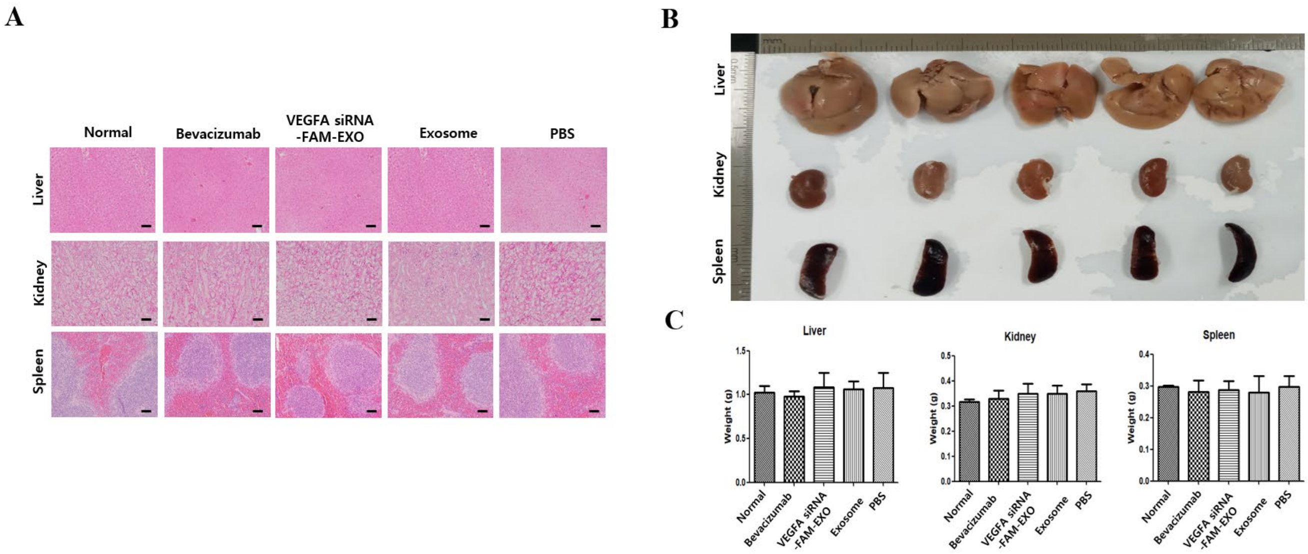


**Figure S3.** Systemic safety evaluation of VEGFA siRNA-FAM-loaded exosomes in mice. (**A**) H&E staining images of the liver, kidney, and spleen. No significant pathological findings, such as inflammatory infiltration, necrosis, hemorrhage, or fibrosis, were observed in the Normal, Bevacizumab, VEGFA siRNA-FAM-EXO, Exosome, or PBS groups. Scale bar = 200 μm. (**B**) Macroscopic images of organs (liver, kidney, and spleen) at the time of sacrifice. No abnormalities, such as liver hypertrophy/atrophy, discoloration, or hemorrhage, were observed in any group. (**C**) Quantification of organ weights (mean ± standard error, n = 6). There were no significant differences in liver, kidney, and spleen weights between groups. Statistics were analyzed using one-way ANOVA followed by Tukey's multiple comparison test.
